# Sorrel Extract Reduces Oxidant Production in Airway Epithelial Cells Exposed to Swine Barn Dust Extract In Vitro

**DOI:** 10.1155/2019/7420468

**Published:** 2019-08-01

**Authors:** Carresse L. Gerald, Chakia J. McClendon, Rohit S. Ranabhat, Jenora T. Waterman, Lauren L. Kloc, Dawn R. Conklin, Ke'Yona T. Barton, Janak R. Khatiwada, Leonard L. Williams

**Affiliations:** ^1^Department of Animal Sciences, North Carolina Agricultural and Technical State University, 1601 East Market Street, Greensboro, NC, USA; ^2^Department of Energy and Environmental Systems, North Carolina Agricultural and Technical State University, 1601 East Market Street, Greensboro, NC, USA; ^3^Center of Excellence for Post-Harvest Technologies, North Carolina Agricultural and Technical University, 500 Laureate Way, Kannapolis, NC, USA

## Abstract

Exposure to hog barn organic dust contributes to occupational lung diseases, which are mediated by inflammatory and oxidative stress pathways. Isoprostanes—a family of eicosanoids produced by oxidation of phospholipids by oxygen radicals—are biomarkers of pulmonary oxidative stress. Importantly, 8-isoprostane has been implicated as a key biomarker and mediator of oxidative stress because it is a potent pulmonary vasoconstrictor. Antioxidants found in fruits and vegetables hold promise for preventing or reducing effects of oxidative stress-related diseases including chronic bronchitis and chronic obstructive pulmonary disease (COPD). Here, we investigated 8-isoP and oxidant production by organic dust-exposed airway epithelial cells and the inhibitory effects of an extract from calyces of the sorrel plant, *Hibiscus sabdariffa*, on oxidant-producing pathways. Confluent cultures of normal human tracheobronchial epithelial cells were pretreated or not with 1% sorrel extract prior to 5% dust extract (DE) exposure. Following DE treatments, live cells, cell-free supernatants, or cell extracts were evaluated for the presence of 8-isoprostane, superoxide, hydrogen peroxide, nitric oxide, hydroxyl radical, peroxynitrite, and catalase activity to evaluate sorrel's inhibitory effect on oxidative stress. The well-known radical scavenging antioxidant, N-acetyl cysteine (NAC), was used for comparisons with sorrel. DE exposure augmented the production of all radicals measured including 8-isoprostane (*p* value < 0.001), which could be inhibited by NAC or sorrel. Among reactive oxygen and nitrogen species generated in response to DE exposure, sorrel had no effect on H_2_O_2_ production and NAC had no significant effect on NO^·^ production. The observations reported here suggest a possible role for sorrel in preventing 8-isoprostane and oxidant-mediated stress responses in bronchial epithelial cells exposed to hog barn dust. These findings suggest a potential role for oxidative stress pathways in mediating occupational lung diseases and antioxidants within sorrel and NAC in reducing dust-mediated oxidative stress within the airways of exposed workers.

## 1. Introduction

Respiratory diseases such as asthma, chronic bronchitis, and chronic obstructive pulmonary disease (COPD) are known to increase with concentrated animal feeding operation (CAFO) exposures [1]. Exposure to animal husbandry dusts, such as organic dust from swine production buildings, is the leading cause of pulmonary disease in these professions. Data collected on farm workers from Iowa and North Carolina from 2005 to 2010 reported an increase of wheezing, coughing, and excessive phlegm [2]. Rodriquez et al. observed that California farmers that worked more years had a higher likelihood of a worse forced expiratory volume (FEV) in 1 second divided by FEV in 6 seconds (FEV_1_/FEV_6_) ratio [3]. Current therapeutic measures do not adequately address symptoms in this population [4].

Many of these occupational lung diseases are mediated by proinflammatory cytokines including interleukin-6 (IL-6) and IL-8, whose levels are known to increase in respiratory cells and tissues following swine dust exposure [5]. IL-8 is a potent recruiter of neutrophils and other granulocytes, and it promotes phagocytosis, a process that contributes to oxidative stress by releasing free radicals to neutralize bacteria. Regardless of the source, free radicals mediate injury by binding to and inhibiting the function of important macromolecules such as proteins, lipids, and DNA. Isoprostanes are prostaglandin-like compounds formed by reaction of free radicals with arachidonic acid in phospholipids and are recognized as biomarkers of oxidative stress [6–9]. Measurement of isoprostanes in various biological fluids is used for assessing oxidative stress in healthy subjects and patients with respiratory diseases including asthma and COPD [10–12]. Inhibition of oxidative stress mechanisms may reduce subsequent airway inflammation.

Phytonutrients are bioactive compounds found in plants that promote health and strengthen immunity [13]. Studies that analyze the anti-inflammatory properties of dietary products and their effects on hog barn dust-mediated inflammation have been conducted. Vitamin D was reported to inhibit organic dust-induced proinflammatory cytokines *in vitro* (human airway epithelial cells and monocytes) and *ex vivo* (mouse lung slices) [14]. Mice exposed to hog barn dust but allowed to consume Moringa tea had fewer white blood cells present in bronchoalveolar lavage than mice only drinking water [15]. *Hibiscus sabdariffa* (sorrel) of the Malvaceae family is a plant native to the West Indies, Jamaica, and China and has been grown in the United States. Sorrel is consumed in foods such as tea, jams, and jellies worldwide [16]. Sorrel, also known as roselle, has been studied for its antimicrobial, anti-inflammatory, and antioxidant capacity [17–20]. Sorrel calyces contain potent phytonutrients that are rich in antioxidants and contain high anthocyanin levels [21].

The purpose of this study was to determine if pretreatment with sorrel will reduce swine facility dust-mediated oxidative stress. Primary airway tracheobronchial epithelial cells were exposed to a dust extract in vitro, and endpoint assays measured production of intracellular oxygen radicals and detection of oxidative stress biomarkers by airway epithelial cells in vitro. Results of our studies showed that pretreatment with sorrel reduced free radical production by airway cells that were exposed to swine facility dust extract. Sorrel's antioxidant action was similar to the well-known radical scavenger, NAC. Taken together, these data reveal previously uncharacterized antioxidant properties of sorrel on airway cells exposed to swine facility dust extract and provide support for future in vitro studies to test effectiveness of sorrel as a potential dietary supplement to prevent swine dust-mediated lung inflammation.

## 2. Materials and Methods

### 2.1. Cell Culture

Normal human bronchial epithelial (NHBE) cells (CC-2541, Lonza, Walkersville, MD) were cultured in NHBE complete media (1 : 1 mixture of BEBM/DMEM, Lonza SingleQuots®, and Nystatin) and maintained in incubators at 37°C with a humidified air conditioner with 5% CO_2_. Cells were used at passage 2 and seeded on rat tail collagen-coated tissue culture plates at a density of 1.0 × 10^4^ cells/cm^2^ or until confluent. Prior to experimental investigations and after cells reached optimal confluency, cells were switched to a nonstimulatory medium that did not contain epidermal growth factor (EGF-NHBE) or serum overnight (i.e., 23 hours ahead of pretreatments). Antioxidant pretreatments were for one hour as described below.

### 2.2. Sorrel Phenolic Extraction and Pretreatment

**Figure 1:**
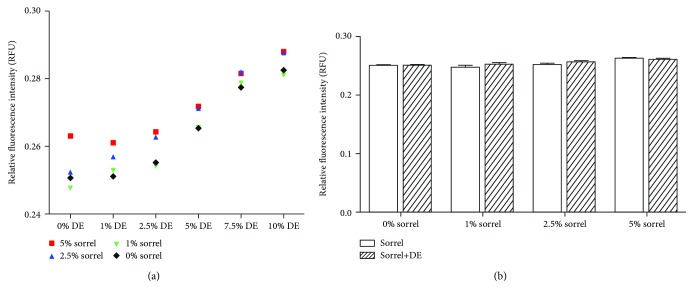
Sorrel and DE dose-response curves. (a) Intracellular ROS production by NHBE cells that were pretreated with sorrel (0-5%) for one hour followed by a 1-hour DE (0-10%) exposure was measured using a DCF assay. 0% DE represents NHBE cells pretreated with 0% sorrel (media), 1% sorrel, 2.5% sorrel, or 5% sorrel only. (b) Intracellular ROS production by NHBE cells pretreated with sorrel (0-5%) for one hour and exposed to 1% DE for one hour. Data are presented as the mean for (a) and the mean ± SEM for (b).

Dried sorrel calyces were purchased from a local grocery store and freeze-dried. The freeze-dried calyces were grounded to powder and then stored at -20°C until use. The phenolics in powdered freeze-dried calyces were methanol extracted by the ultrasound-assisted method as was previously described [22]. To determine the optimal dose of sorrel to be used with dust extract (DE), NHBE cells were incubated with 0-5% sorrel phenolic extracts diluted in culture media for one hour followed by a 1-hour exposure to DE (0-10%). ROS generation in response to sorrel and/or DE gradients was measured using the 2′,7′-dichlorofluorescein diacetate (DCF) assay (Molecular Probes, Eugene, OR), according to the manufacturer's instructions. Briefly, NHBE cells were loaded with 10 *μ*M DCF in phenol red-free media for 50 minutes at 37°C, rinsed twice with phosphate-buffered saline, and treated as described. DCF fluorescence was read at 485 nm excitation and 538 emission.  Figure 1 summarized dose-response curves and indicates that lower sorrel concentrations (i.e., 1% or 2.5%) did not increase ROS generation when combined with DE (Figure 1(a)). As shown in Figure 1(b), when NHBE cells were pretreated with 0-5% sorrel for one hour prior to exposure to 1% DE for one hour, there was not a significant increase in ROS. Therefore, the sorrel phenolic extract was diluted to 1% sorrel in cell culture medium for a one-hour pretreatment prior to dust extract exposure.

### 2.3. N-Acetyl-L-cysteine (NAC) Pretreatment

NAC is a potent free radical scavenging compound with antioxidant activity. For pretreatments, 20 mM NAC (Sigma-Aldrich, St. Louis, MO) was prepared in culture media and used to pretreat cells for one hour. This concentration was chosen based on a study from Ye et al. [23], where the authors utilized a 20 mM NAC concentration to decrease nanotube-mediated ROS generation in A549 lung epithelial cells [23].

### 2.4. Preparation of Swine Confinement Facility Dust Extract (DE)

Settled dust was collected from raised surfaces within the swine confinement facility at North Carolina A&T State University, and DE was prepared by dissolving dust (1 g) in HBSS, centrifugation, and 0.2 *μ*m filtration as was previously described [24].

### 2.5. DE Exposure and Cell-Free Supernatant Collection

Confluent cultures of NHBE cells that were previously switched to nonstimulatory media for 23 hours then pretreated with sorrel or NAC or kept in media for one hour were washed twice with PBS, then stimulated with DE for 7 hours or as indicated. Post DE exposure, supernatant was collected and centrifuged for 5 minutes at 5,000 RPM. After centrifuging, the supernatant was transferred to a clean microcentrifuge tube without disturbing the cell pellet. The supernatants were kept at -20°C (i.e., for 8-isoprostane, ROS). Supernatants were used immediately upon acquisition for NO^·^ assays.

### 2.6. Measurement of Cell Viability

To determine the cytotoxic effect of the agricultural dusts on the NHBE cells, a lactate dehydrogenase (LDH) assay (Roche Diagnostics) was performed as per the manufacturer's instructions on the cell-free supernatant. Cell viability was also assessed using a LIVE/DEAD assay (Molecular Probes) according to the instruction manual.

### 2.7. Proliferation Assay

Cells were cultured in a clear 96-well plate until they reached 75% confluency. Cells were then pretreated or not with either NAC or sorrel, stimulated with DE, and analyzed using the bromodeoxyuridine/5-bromo-2′-deoxyuridine (BrdU) cell proliferation assay (Roche Diagnostics) as per the manufacturer's instructions.

### 2.8. Measurement of Superoxide Production by Mitochondria

MitoSOX Red permeates live cells, selectively targets mitochondria where superoxide (O_2_^·-^) are generated, but not other ROS, and rapidly oxidizes them into a highly fluorescent product [25]. Cells were pretreated or not with antioxidants for one hour and DE for 7 h, washed, and stained with MitoSOX Red at 37°C for 60 minutes, and then, O_2_^·-^ action within cells was measured within 3 hours at 37°C. Cells were imaged using an EVOS FL microscope (AMG) according to the manufacturer's instructions. Relative fluorescence intensity was measured using ImageJ software.

### 2.9. Detection of Hydrogen Peroxide, Hydroxyl Radical, and Peroxynitrite

To measure production of reactive molecules hydroxyl radical (^·^OH) and peroxynitrite (ONOO^−^) produced by DE-exposed cells, a hydroxyphenyl fluorescein (HPF) assay kit was used. For hydrogen peroxide (H_2_O_2_) and peroxidase activity assessment, the Amplex Red assay was used. For both assays, the procedures and excitation and emission settings directed by the manufacturer (Molecular Probes) were followed. ImageJ software was used to measure fluorescence intensity.

### 2.10. Nitric Oxide Production

To measure nitric oxide (NO^·^) production, cell-free supernatants from exposed cells were evaluated using a Griess reagent assay (Promega) according to the manufacturer's instructions.

### 2.11. Measurement of Catalase Activity

To measure catalase activity in the NHBE cell cultures exposed to DE in combination with antioxidant pretreatments or none, a catalase activity assay was performed according to the manufacturer's instruction (Cayman Chemical, Ann Arbor, Michigan) and absorbance was read at 540 nm using a SpectraMax M5 microplate reader.

### 2.12. Measurement of 8-Isoprostane

To evaluate oxidative stress, an 8-isoprostane (8-isoP) EIA kit (Cayman Chemical, Ann Arbor, MI) was used on cell supernatants of exposed cells as per the manufacturer's instructions. Supernatants were either diluted 1 : 10 or left undiluted.

### 2.13. Statistical Analysis

For cell viability studies (LDH and proliferation), one-way analysis of variance followed by Bonferroni posttest for normally distributed data was used. To determine the effect of antioxidant pretreatment on oxidant production following DE exposure, two-way analysis of variance with Bonferroni posttest correction for normally distributed data was used to compare means of control/treatment groups (i.e., media only or DE exposure) and antioxidant groups (i.e., antioxidants with media or antioxidants with DE). Each experiment was conducted at least three independent times (*n* = 3). For all analyses, a two-sided significance level of 0.05 was used.

## 3. Results

### 3.1. Effect of Sorrel Phenolic Extracts on ROS Generation by NHBE Cells

To determine the concentration of sorrel phenolic extract for use in combination with DE, NHBE cells were incubated with 0-5% sorrel for one hour followed by exposure to DE (0-10%) for one hour. Since this study focused on oxidant production, we were interested in knowing if sorrel would induce ROS production. Therefore, NHBE cells were loaded with the nonfluorescent cell-permeant 2′,7′-dichlorodihydrofluorescein diacetate, which becomes the highly fluorescent DCF compound when the acetates are cleaved by intracellular esterases and oxidation. Lower concentrations of sorrel phenolic extracts, specifically 1% and 2.5%, did not cause ROS generation by NHBE cells exposed to sorrel only or cells pretreated with sorrel and exposed to increasing concentrations of DE (Figures 1(a) and 1(b)). However, increasing DE concentration caused a dose-dependent increase in ROS production by NHBE cells. It was determined that, when combined, lower concentrations of sorrel (1%) and DE (1%) would provide adequate information about DE-regulated ROS production by NHBE cells. While the DCF assay is a good measure for overall ROS production, additional studies were conducted with assays specific for various types of ROS; results are described below.

### 3.2. Cytotoxicity Levels Increased in NHBE Cells following Exposure to Swine Barn Dusts

**Figure 2:**
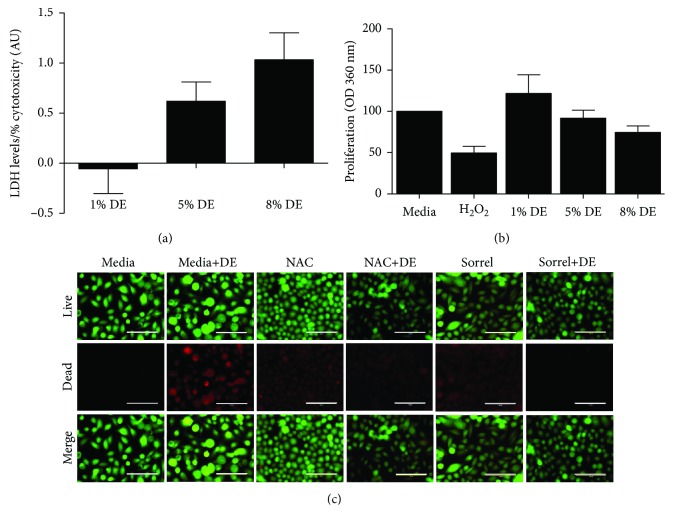
Effect of DE exposure on LDH release, cell proliferation, and viability of NHBE cells. (a) LDH activity within supernatant after 6-hour exposure to 1-8% DE, *n* = 3. (b) Cell proliferation (BrdU assay) after 24-hour exposure to 1-8% DE, *n* = 4. (c) Representative images of LIVE/DEAD staining to assess cell viability after exposure to 5% DE for 7 hours, *n* = 3. The green fluorescence (i.e., GFP) and red fluorescence (i.e., RFP), respectively, represent live cells stained with calcein and dead cells stained with ethidium homodimer. Data are presented as the mean ± SEM for (a) and (b).

Lactate dehydrogenase (LDH) is an enzyme that normally resides in the cytoplasm of the cell. When the plasma membrane is damaged, LDH can be released into the surrounding environment. This leakage can then be used as a marker of cell viability since the level of LDH in culture media is directly proportional to the level of cytotoxicity. In this study, cytotoxicity levels of DE were evaluated and the results are shown in Figure 2(a). A dose-dependent increase of LDH release was observed in cells exposed to hog barn 1-8% DE.

### 3.3. Effect of DE Exposure on Cell Viability and Proliferation

To assess cell vitality during experimental treatments, viability and proliferative ability assessments were conducted during treatments with live cells. Lower DE concentrations (i.e., 1-5%) did not alter proliferation. However, Figure 2(b) showed higher DE levels—8%—which resulted in a 25% reduction in cell proliferation compared to the control, although this did not reach formal significance.  Figure 2(c) shows cells stained with LIVE/DEAD stain and DAPI. The treatment conditions did not affect cell viability as denoted by the limited number of red (i.e., RFP) fluorescent cells. Thus, the 5% DE concentration was selected for subsequent experiments because it initiated a response, i.e., LDH release, in cells that was nontoxic, i.e., no appreciable decrease in cell proliferation or cell viability.

### 3.4. Hog Barn Dust Increases ROS and RNS Production by NHBE Which Is Reduced by Sorrel

**Figure 3:**
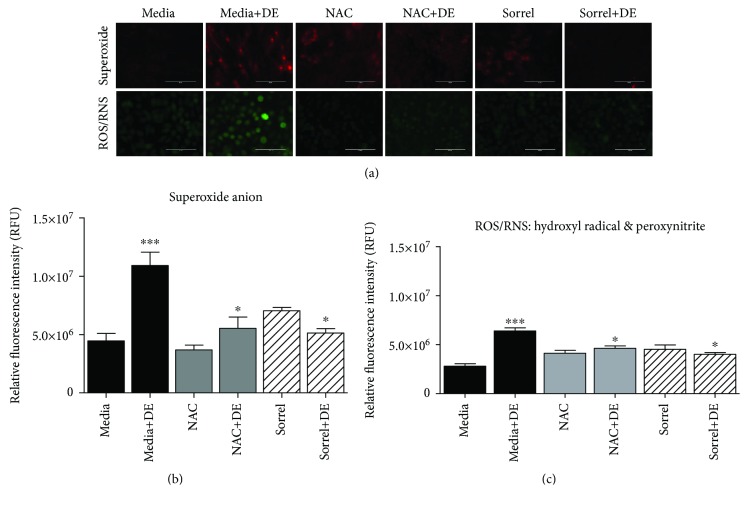
DE-mediated increase of ROS/RNS production by NHBE cells is inhibited by sorrel and NAC. (a) NHBE cells were treated with media, media+5% DE, sorrel, sorrel+5% DE, NAC, and NAC+5% DE for seven hours. After which, cells were loaded with dye to detect mitochondrial superoxide anion or cytosolic ROS/RNS for 1 hour, and oxidant production by live cells was measured via fluorescent microscopy within 1-2 hours post staining. A representative image of 4-5 images per treatment is shown. (b) Relative fluorescence intensity as determined by ImageJ for mitochondrial superoxide anion detected in (a). (c) Relative fluorescence intensity for hydroxyl radical and peroxynitrite as determined by ImageJ for hydroxyl radical and peroxynitrite detected in (a). ^∗∗∗^*p* < 0.001 compared to media. ^∗^*p* < 0.05 compared to media+DE. Data are presented as the mean ± SEM for (b) and (c).

To further determine the effect of animal husbandry dusts on cellular responses, intracellular production of peroxynitrite and hydroxyl radical accumulation were examined. In Figure 3, NHBE cells were exposed to 5% DE for seven hours following pretreatment with media, 20 mM NAC, or 1% sorrel, then stained with MitoSOX to detect superoxide or HPF to detect ROS/RNS (i.e., hydroxyl radical and peroxynitrite). As shown in Figure 3(a), mitochondrial superoxide generation increased after hog barn DE exposure, which was reduced by sorrel and NAC.  Figure 3(b) showed that fluorescence intensity of elevated superoxide levels following 5% DE exposure (*p* < 0.001) was significantly reduced by both NAC and sorrel pretreatments (*p* < 0.05).  Figure 3(c) showed fluorescence intensity for DE-mediated intracellular production of hydroxyl radical and peroxynitrite (*p* < 0.001). Pretreatment with sorrel and NAC decreased the DE-mediated superoxide, hydroxyl radical, and peroxynitrite production (*p* < 0.05).

### 3.5. Pretreatment with Sorrel Attenuates NO^·^ Levels in DE-Exposed NHBE Cells

**Figure 4:**
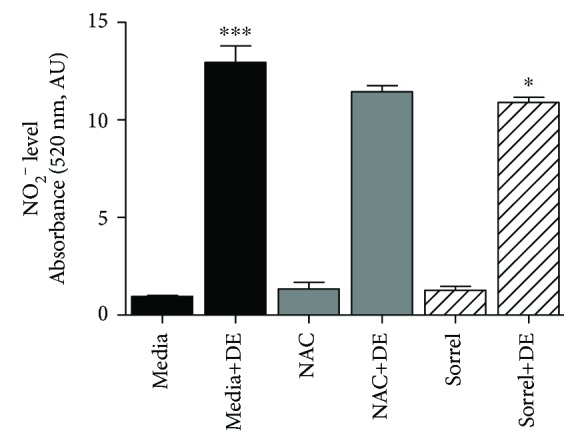
Enhanced nitric oxide secretion by cultures of human airway epithelial cells following DE exposure. NHBE cells were pretreated with 20 mM NAC or 1% sorrel followed by a 7-hour 5% DE exposure, and NO_2_^−^ levels were measured in cell-free supernatant using the Griess reagent. ^∗^*p* value < 0.05 compared to DE. ^∗∗∗^*p* value < 0.001 compared to media. Data are presented as the mean ± SEM, *n* = 3.

Nitric oxide is a free radical that is predominant in the respiratory tract and is normally found as a signaling molecule. In this study, the levels of nitric oxide in cells exposed to DE as well as an antioxidant pretreatment of NAC or sorrel were examined. In Figure 4, NHBE cells were pretreated with antioxidants (one hour) prior to 5% swine DE exposure for 7 hours. The 5% DE exposure increased nitric oxide levels compared to the control (*p* < 0.001). Sorrel pretreatment had low supernatant levels of nitric oxide compared to media+DE-exposed cells whereas NAC did not decrease nitric oxide levels.

### 3.6. Hog Barn Dust Mediates an Increase of Hydrogen Peroxide in NHBE

**Figure 5:**
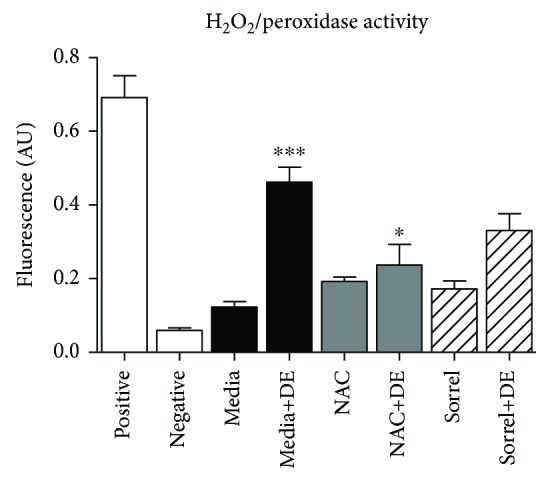
Hydrogen peroxide generation increased in cells exposed to DE. NHBE cells were pretreated or not with 20 mM NAC or 1% sorrel and kept in media or exposed to DE for seven hours. After the treatment, the supernatant was collected for measurement of hydrogen peroxide/peroxidase activity using the Amplex Red reagent. ^∗∗∗^*p* < 0.001 vs. media. ^∗^*p* < 0.05 vs. media+DE. Data are presented as the mean ± SEM, *n* = 3.

To further delineate the ROS involved in hog barn dust inflammation, we stimulated NHBE with 5% DE, with or without sorrel and NAC pretreatment, and with 300 *μ*M hydrogen peroxide (H_2_O_2_) as a positive control.  Figure 5 demonstrates DE-induced hydrogen peroxide/peroxidase activity in NHBE cells (*p* < 0.001). NAC reduced hydrogen peroxide/peroxidase activity (*p* < 0.05); however, sorrel do not affect the hydrogen peroxide/peroxidase activity.

### 3.7. Catalase Activity Was Increased by DE Exposure

**Figure 6:**
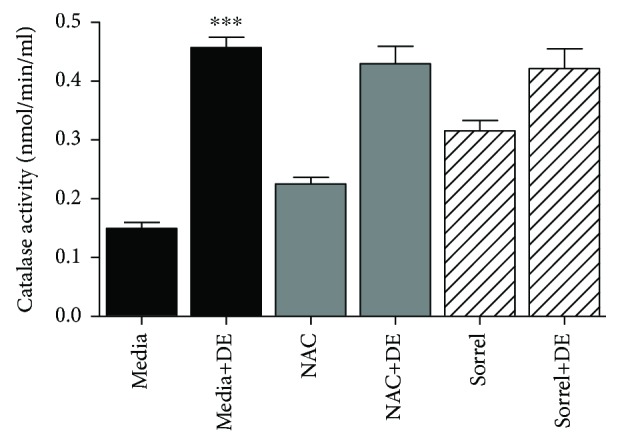
Catalase activity is enhanced in NHBE cells exposed to DE. NHBE cells were exposed to media, media+5% DE, NAC, NAC+5% DE, sorrel, and sorrel+5% DE (7 h). After exposure, cells were lysed and protein was extracted. Catalase activity was measured via its reaction rate with hydrogen peroxide. ^∗∗∗^*p* < 0.001 compared to media. Data are presented as the mean ± SEM, *n* = 3.

Catalase is an endogenous enzymatic antioxidant that catalyzes the conversion of H_2_O_2_ to molecular oxygen and water. Therefore, we were interested to know whether sorrel had an effect on enhancing catalase activity in DE-exposed cells. In Figure 6, NHBE were exposed to media only or 5% DE, with or without NAC (20 mM) and sorrel (1%) pretreatments. Catalase activity was increased in cells exposed to 5% DE compared to media and also sorrel-pretreated cells compared to media (*p* < 0.001). NAC and sorrel did not affect catalase activity when used as pretreatments prior to DE exposure.

### 3.8. Levels of 8-Isoprostane Are Increased by Swine Dust, and Sorrel Attenuates 8-Isoprostane Levels

**Figure 7:**
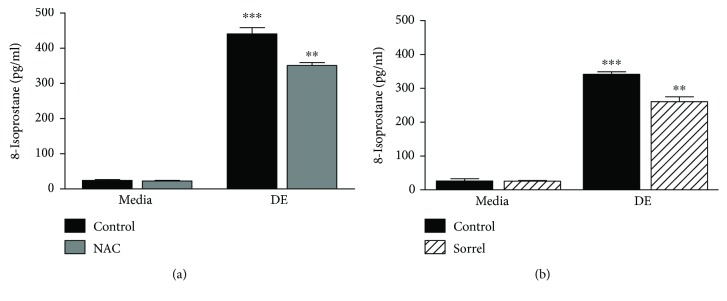
Pretreatment with sorrel attenuates 8-isoprostane release by NHBE cells exposed to DE. Pretreatment of NHBE cell cultures with 20 mM NAC (a) or 1% sorrel (b) limited plasma membrane damage—as measured by diminished 8-isoprostane release—following 5% DE exposure for 7 hours. ^∗∗^*p* value < 0.01 compared to media+DE. ^∗∗∗^*p* value < 0.001 vs. media. Data are presented as the mean ± SEM, *n* = 3.

The eicosanoid, 8-isoprostane, is a member of the arachidonic acid pathway and is a well-known mediator of oxidative stress [26]. Therefore, since 8-isoprostane is commonly found in the airway and is a known biomarker for oxidative stress, levels of 8-isoprostane were evaluated in NHBE cells that had been exposed to DE for 7 hours following a one-hour pretreatment with 20 mM NAC or 1% sorrel. The levels of 8-isoprostane were significantly higher in the DE only-treated cells as depicted in Figures 7(a) and 7(b). The antioxidant pretreatments with NAC and sorrel significantly lowered 8-isoprostane levels in DE-exposed cells in a similar manner (*p* < 0.05).

## 4. Discussion

Hog barn dust exposure induces a myriad of complications on the respiratory epithelium, and facility workers are at risk for development of respiratory symptoms and disease [1]. In Pender et al. [24], results show that hog barn dust (5 and 10%) induces ROS in a dose-dependent manner in THP-1 cells. Wyatt et al. reported that hog barn dust increased nitric oxide levels in a time-dependent manner in bovine ciliated cells [27]. In our current study, we investigated similar effects of hog barn dust, seeking to define the oxidant species produced and also in the context of reversing the ensuing oxidative stress with naturally occurring antioxidants. Many phytonutrients have immunomodulatory effects, and consuming a diet of foods rich in antioxidants can be beneficial for these types of occupational respiratory exposures. Calyces of *Hibiscus* spp. contain potent free radical scavenging antioxidants such as polyphenolic acids, flavonoids, and anthocyanins [22]. In this study, we utilized a 5% hog barn dust extract similar to findings by Wyatt et al. [27]. Due in part to the complexity of the dust such as the composition of nanoparticles, endotoxins, and other biophysical components, we observed various types of oxidants being induced [28]. With this knowledge of sorrel's ability to protect respiratory defenses, we tested our hypothesis that polyphenolic sorrel extracts alleviate hog barn dust-mediated oxidant levels.

Our current investigations indicate that hog barn dust increases oxidant species and these oxidants can be quenched by the use of antioxidants. Upon noting that lactate dehydrogenase levels increase with increasing hog barn dust extract concentration, we conducted fluorescence assays that will identify several reactive oxygen and nitrogen species in the hog barn dust extract-exposed cells. We observed an increase of ROS/RNS in cells exposed to 5% hog barn dust for seven hours. Interestingly, NAC and the *Hibiscus sabdariffa* (sorrel) polyphenolic extract demonstrated similar reducing actions on oxidant production by DE-exposed NHBE cells. The commercially available antioxidant, acetylcysteine (also known as N-acetyl cysteine, N-acetyl-L-cysteine, or NAC), is a thiol compound that has mucolytic potential and is a direct precursor to reduced glutathione (antioxidant) [29]. NAC is also known to decrease respiratory bursts caused by neutrophils, which can lead to a decrease in free radicals and ultimately reduce oxidative stress [30, 31].

This data is consistent with Pender et al., which demonstrates that hog barn dust induces ROS *in vitro* [24]. We demonstrate that lactate dehydrogenase (LDH) levels increase in a dose-dependent manner which complements the data that shows that hog barn dust induces 8-isoprostane, a biomarker of lipid peroxidation. Free radicals steal electrons from lipids found in the cell membrane which in turns damages the cell causing LDH to seep out. The 8-isoprostane molecule is known to regulate human airway smooth muscle function and can be upregulated in airway diseases such as asthma as well as environmental exposure [32]. Asthmatic children exhibit higher levels of isoprostanes in their exhaled breath condensate [33]. In the study completed by Janicka et al., there were increased levels of 8-isoprostanes in patients with pulmonary diseases such as asthma and chronic bronchitis compared to healthy individuals [34]. Anokwuru and colleagues reported that phenolic extracts were also capable of inhibiting lipid peroxidation [35]. Nordgren and colleagues provided evidence that maresin-1 could decrease levels of organic dust-induced production of IL-8 and IL-6 by bronchial epithelial cells [36]. Maresin-1 can function as a lipid mediator that can alleviate oxidative stress. Anthocyanins also are capable of antioxidative activity against low-density lipoprotein (LDL) oxidation in RAW264.7 cells [35]. Our 8-isoprostane data are consistent with our observations which show that cells exposed to 8% hog barn dust extract experience reduced proliferation, although not significantly. Cells exposed to higher concentrations of dust could be undergoing too much stress to keep up regular maintenance such as proliferation. Losing cells to lipid peroxidation damage could result in general tissue damage due to cells reducing proliferation and cell death.

To answer which free radical species may have played a role in cell damage, we probed several ROS targets. We observed increased hydrogen peroxide levels with a 5% hog barn dust extract exposure, which lead us to suggest that hydrogen peroxide is acting on the cell membrane and causing the increase of LDH observed in this study. Sorrel extracts did not provide protection against hydrogen peroxide for cells exposed to 5% hog barn dust extract. However, NAC, the commercially available antioxidant used for comparisons, did reduce hydrogen peroxide levels. Due to the common cascade of events in the cell, superoxide dismutase will partition superoxide which results in the formation of hydrogen peroxide. Knowing this, we examined the mitochondrial superoxide anion levels. As expected, 5% hog barn dust extract increased the levels of superoxide in NHBE cells. Sorrel was able to reduce superoxide levels at a level similar to NAC. This is significant because it demonstrates a window that sorrel would be able to provide protection. The modulation of the free radical nitric oxide or NO^·^ was also evaluated. NO^·^ acts as a signaling molecule within the airway and can mediate ciliary beat frequency [37]. We observed increases of NO^·^ in response to 5% DE exposure *in vitro*. This result falls in line with findings from Gerald et al., that NF-*κ*B activity is increased in BEAS2B cell cultures with a 5% hog barn dust exposure [5]. NF-*κ*B is an upstream transcription factor that codes for the gene inducible nitric oxide synthase (iNOS). It is speculated that hog barn dust-mediated ROS contribute to activation of pathways leading to the translocation of NF-*κ*B since it has been reported that reactive oxygen intermediates can stimulate NF-*κ*B signaling [38]. Pretreatment with NAC did not significantly decrease the levels of NO^·^; however, sorrel attenuated these effects. NO^·^ is also a known regulator of ciliary beating. In Wyatt et al. [27], exposure to hog barn dust extract altered epithelial beating in bovine ciliated cells in vitro. More studies are warranted to fully understand the significance of oxidants such as NO^·^ in airway cells since NO^·^ is a potent signaling molecule. For example, it is responsible for mediating relaxation of airway smooth cells by modulating calcium [39], an important signaling molecule and cofactor for numerous enzymatic reactions. Thus, it is plausible that in this study, increased levels of NO^·^ released by agricultural dust-stimulated airway epithelial cells could cause a beneficial effect by promoting the relaxation of airway smooth muscle cells; however, this effect was not investigated.

NO-derived compounds including peroxynitrite are known to cause inflammation, oxidative stress, and activation of metalloproteases and inactivation of antiprotease in COPD. Superoxide rapidly couples with NO^·^ to produce the potent proinflammatory molecule peroxynitrite [40, 41]. The endogenous enzymatic antioxidant SOD partitions superoxide into molecular oxygen or hydrogen peroxide which limits its availability for reacting with NO^·^ and other molecules. However, peroxynitrite formation occurs under normal physiological conditions and in the presence of SOD because the reaction of superoxide with NO^·^ occurs more rapidly than the rate of superoxide dismutation [42]. Peroxynitrite is believed to be responsible for most of the toxic actions of superoxide and NO^·^ [43]. In the present study, we observed enhanced peroxynitrite and hydroxyl radical formation by DE-exposed NHBE cells, which was prevented by sorrel and NAC. This finding is consistent with observations reported by Fischer and Voynow, where pre- or coincubation of A549 cells and NHBE cells with dimethylthiourea (DMTU), a scavenger of peroxynitrite, hydroxyl radical, and other hydroxylated products, reduced neutrophil elastase-induced MUC5AC gene expression and oxidative stress [44].

An antioxidant-rich diet can provide benefits such as decreasing free radical levels and supplying the body with free radical eliminators. The pretreatment of NHBE cells with sorrel reduced NO^·^ in the sorrel and DE-treated cultures which suggests a protective effect [45]. The phenolic compounds that can be found in sorrel are beneficial to the plant to ward against fungi, viruses, and bacteria. These compounds can also be beneficial to humans; phenols in particular are known to scavenge radicals [46]. The sorrel components could be extremely helpful for inhibiting bacterial growth [17]. Since humans cannot synthesize naturally occurring antioxidants like polyphenols, obtaining them from a diet rich in plants such as sorrel can be advantageous [46, 47]. The sorrel extract that is rich in polyphenols has been reported to be more effective than vitamin C to ward off oxidative stress in tissues [39]. The levels of phenolics are higher than many other active substances from plant products and can be efficient as a cytoprotectant, anti-inflammatory and antibacterial [46, 48]. Gerald et al. provide evidence of Listeria, Salmonella, and Escherichia that could be present in the hog barn dust as well as particles that could be deposited in the airways [28]. The exogenous antioxidant components such as manganese and ascorbic acid are found in the sorrel calyx and could help protect against inflammation and other diseases [49]. The sorrel plant as a whole has the potential to be a beneficial antioxidant source. Keeping organic dust components in mind, the polyphenols within sorrel could be participating in antimicrobial and anti-inflammatory mechanisms, although those actions were not investigated here.

As we expected, catalase activity levels increased when cells were exposed to hog barn dust extract and was consistent with elevated hydrogen peroxide levels when cultured cells were treated with hog barn dust extract. We did not observe significant changes in DE-induced catalase activity when cells were exposed to NAC or sorrel in combination with 5% hog barn dust extract exposure, suggesting that sorrel and NAC may not be interfering with catalase activity. We observed an increase of catalase activity in cells exposed to the polyphenolic compounds from sorrel which is consistent with other reports where antioxidant supplements, such as N(G)-nitro-L-arginine methyl ester (L-NAME), increased activities of catalase and SOD [50]. This data represents the antioxidant role of the extract and, more specifically, the polyphenolic extract containing compounds that encourage antioxidant production. In future studies, longer exposure times (beyond 7 hours) will be used to observe levels of catalase and its return to basal levels post hog barn dust extract exposure. Furthermore, studies aimed at assessing the mechanism of airway inflammation induced by hog barn dust exposure and the anti-inflammatory effects of polyphenolic extracts should also take into consideration and measure neutrophil chemotactic mediators including leukotriene B4 (LTB4) [51].

## 5. Conclusions

In summary, exposure to hog barn dust can increase levels of oxidants in respiratory cells. Our findings suggest that hog barn dust modulates oxidant and antioxidant levels in airway epithelium. We have shown that hog barn dust exposures can intensify oxidant levels, and polyphenolic extracts from *Hibiscus sabdariffa* can mitigate these levels. We report for the first time that hog barn dust extract mediated superoxide, hydrogen peroxide, and lipid peroxidation in human-derived tracheobronchial epithelium and is reduced by pretreatment with a polyphenolic extract of sorrel calyces. Future studies are warranted to further investigate the molecular mechanisms associated with the regulatory effects of hog barn dust on antioxidants such as catalase. Such observations may have imperative consequences on the health of agricultural workers exposed to hog barn dust, and these data present a possible natural solution for reduction of oxidants and subsequent inflammation.

## Data Availability

The data used to support the findings of this study are available from the corresponding author upon request.

## Abbreviations

8-isoP: 8-Isoprostane
ANOVA: Analysis of variance
BrdU: Bromodeoxyuridine, 5-bromo-2′-deoxyuridine
CAFO: Concentrated animal feeding operations
COPD: Chronic obstructive pulmonary disease
DAPI: 4′,6-Diamidino-2-phenylindole
DCF: 2′,7′-Dichlorofluorescein
DE: Dust extract
DMTU: Dimethylthiourea
EGF: Epidermal growth factor
FEV_1_/FEV_6_: Forced expiratory volume (FEV) in 1 second divided by FEV in 6 seconds
GFP: Green fluorescent protein
HPF: Hydroxyphenyl fluorescein
IL: Interleukin
LDH: Lactate dehydrogenase
LDL: Low-density lipoprotein
L-NAME: N(G)-Nitro-L-arginine methyl ester
MUC5AC: Mucin 5AC
NAC: N-Acetyl cysteine
NF-*κ*B: Nuclear factor kappa B
NHBE: Normal human bronchial epithelial
RAW264.7: Macrophage Abelson murine leukaemia virus transformed
RFP: Red fluorescent protein
RPM: Revolutions per minute
RNS: Reactive nitrogen species
ROS: Reactive oxygen species
SEM: Standard error measure.
